# Vascularized Lung Cancer Model for Evaluating the Promoted Transport of Anticancer Drugs and Immune Cells in an Engineered Tumor Microenvironment

**DOI:** 10.1002/adhm.202102581

**Published:** 2022-03-21

**Authors:** Dasom Kim, Kyeong Seob Hwang, Eun U Seo, Suyeong Seo, Byung Chul Lee, Nakwon Choi, Jonghoon Choi, Hong Nam Kim

**Affiliations:** ^1^ Brain Science Institute Korea Institute of Science and Technology (KIST) Seoul 02792 Republic of Korea; ^2^ School of Mechanical Engineering Yonsei University Seoul 03722 Republic of Korea; ^3^ Division of Bio‐Medical Science and Technology KIST School Korea University of Science and Technology (UST) Seoul 02792 Republic of Korea; ^4^ Program in Nano Science and Technology Graduate School of Convergence Science and Technology Seoul National University Seoul 08826 Republic of Korea; ^5^ KU‐KIST Graduate School of Converging Science and Technology Korea University Seoul 02841 Republic of Korea; ^6^ School of Integrative Engineering Chung‐Ang University Seoul 06974 Republic of Korea; ^7^ Yonsei‐KIST Convergence Research Institute Yonsei University Seoul 03722 Republic of Korea

**Keywords:** angiogenesis, drug delivery, immune cell transport, tumor spheroids, vascularization

## Abstract

The tumor microenvironment (TME) is the environment around the tumor, including blood vessels, immune cells, fibroblasts, signaling molecules, and the extracellular matrix (ECM). Owing to its component interactions, the TME influences tumor growth and drug delivery in a highly complex manner. Although several vascularized cancer models are developed to mimic the TME in vitro, these models cannot comprehensively reflect blood vessel–tumor spheroid interactions. Here, a method for inducing controlled tumor angiogenesis by engineering the microenvironment is presented. The interstitial flow direction regulates the direction of capillary sprouting, showing that angiogenesis occurs in the opposite direction of flow, while the existence of lung fibroblasts affects the continuity and lumen formation of sprouted capillaries. The vascularized tumor model shows enhanced delivery of anticancer drugs and immune cells to the tumor spheroids because of the perfusable vascular networks. The possibility of capillary embolism using anticancer drug‐conjugated liquid metal nanoparticles is investigated using the vascularized tumor model. This vascularized tumor platform can aid in the development of effective anticancer drugs and cancer immunotherapy.

## Graphical abstract

A method for inducing controlled tumor angiogenesis by engineering the microenvironment is presented. In the engineered vascularized tumor microenvironment, the promoted transport of anticancer drugs, immune cells, and embolism materials is shown.

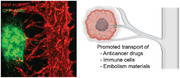

## 1 Introduction

Successful cancer treatment requires innovative drugs and delivery methods; however, the development of such innovative strategies requires a long time and enormous investments.^[^ ^1^, ^2^ ^]^ Over the last few decades, drug development has necessitated the use of multiple screening models, including plate‐cultured cell lines and animal models, depending on the stage of drug development. However, plate‐cultured cells do not exhibit satisfactory drug responsiveness because these models lack multiple important in vivo factors, such as cell–cell interactions and microenvironmental cues. Thus, their utilization has been limited to early toxicity testing. Animal models have shown in vivo drug responses; however, they also have inherent limitations in predicting drug toxicity and efficacy in humans, owing to species differences.^[^ ^3^ ^]^ Currently, the poor predictive ability of preclinical models is a significant challenge. Therefore, the development of predictable biomimetic models using inexpensive and efficient in vitro technologies is actively being studied.^[^ ^4^, ^5^ ^]^

Biomimetic in vitro models can bridge the gap between 2D cultured cells and animal models by mimicking the 3D architecture of in vivo tissues. In the case of 3D in vitro models such as spheroids or organoids, physiological conditions are similar to those of in vivo tissues because the concentration gradients of culture medium, oxygen, and drugs can be mimicked around the cells, and cell–cell interactions are ensured.^[^ ^6^ ^]^ In such a biomimetic environment, cells are expected to exhibit more in vivo‐like responses.^[^ ^7^, ^8^ ^]^ Microphysiological systems, which are in vivo‐resembling cell culture platforms based on microfluidic technology, have proven to be promising for drug screening.^[^ ^9^, ^10^, ^11^, ^12^ ^]^ To reproduce a complex 3D environment in which cell–cell and cell–matrix interactions are possible on a microfluidic chip, 3D cell aggregate models such as spheroids and organoids are cultured with an extracellular matrix (ECM) as a scaffold.^[^ ^13^ ^]^

The microphysiological system is a suitable platform for studying the interactions between tumors and the microenvironment. Recently, to investigate the effects of the tumor microenvironment (TME) on tumor growth, metastasis, and drug sensitivity, tumor spheroids and several stromal cells were cocultured in a hydrogel environment.^[^ ^14^, ^15^, ^16^, ^17^, ^18^, ^19^, ^20^, ^21^ ^]^ In particular, vascular endothelial cells are frequently cultured with tumor spheroids and form a vascular network to deliver nutrients and oxygen, and these vascularized models can be used to test drug delivery systems.^[^ ^22^ ^]^ Previous studies have revealed that multiple factors, including fibroblasts, interstitial flow, and growth factors, can regulate angiogenesis.^[^ ^23^, ^24^, ^25^ ^]^ The existence of lung fibroblasts is one of the key initiating factors of angiogenesis. Since lung fibroblasts produce proangiogenic factors, the position of lung fibroblasts is an important factor in the angiogenic direction.^[^ ^26^ ^]^ The orientation of interstitial flow is also known to regulate the angiogenic direction. In in vivo tumors, their rapid growth induces high interstitial fluid pressure (IFP) around them, thereby generating outward interstitial flow.^[^ ^27^ ^]^ Previous studies have shown that blood and lymphatic vessels grow against the direction of interstitial flow.^[^ ^28^, ^29^ ^]^ In addition to these factors, the types of hydrogel matrix are important. For example, fibrin and collagen type I are known to promote angiogenic sprouting in the presence of flow.^[^ ^30^, ^31^ ^]^

Several approaches have been developed to form blood vessels within microphysiological systems,^[^ ^29^, ^32^, ^33^, ^34^ ^]^ and recent studies have achieved vascular perfusion in the grown microvasculature.^[^ ^35^, ^36^, ^37^, ^38^, ^39^ ^]^ In the case of platforms typically used for modeling vascularized tumors, micropillars, needles, and 3D printing have been used.^[^ ^21^, ^40^, ^41^, ^42^, ^43^ ^]^ The development of these models enables not only research on tumor–TME interactions, but also enables their application in anticancer drug screening.^[^ ^44^ ^]^ However, vascular structures in previous studies were typically not fully embedded in a 3D matrix (e.g., not completely attached to the surface of the gelled hydrogel) and could therefore not exhibit the directional selection of cellular behaviors in a single microphysiological system. Furthermore, even vascularized tumor models, such as the delivery of therapeutic modalities, did not show an effective function in tumor treatment.

Herein, we report methods for controlling angiogenesis in lung cancer spheroids and their application in the efficacy testing of cancer treatment modalities. By controlling the direction of interstitial flow, existence and location of human lung fibroblasts, and location of lung cancer spheroids, we aimed to control the rate and growth direction of tumor angiogenesis in an elaborate manner. In the perfusable vascularized tumor platform, we screened the effects and delivery of anticancer drugs to tumor spheroids with a particular focus on the role of sprouted capillaries. Furthermore, the transport of immune cells through the sprouted capillaries has also been investigated for their potential application in immune cell delivery and embolism of tumor capillaries.

## 2 Results

### 2.1 Development of a Vascularized Lung Cancer Model

**Figure 1:**
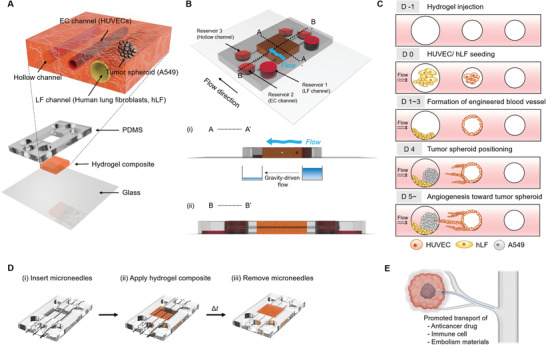
Design of the microfluidic chip for the vascularized tumor model. A) Explosive view of the 3D vascularized lung cancer model within the hydrogel composite. B) Cross‐sectional view of the hydrogel‐laden chip and the induction of interstitial flow. C) Experimental steps in the seeding of cells and induction of angiogenic sprouting by controlling the engineered tumor microenvironment. D) Fabrication steps for microvascular structure embedded in the composite hydrogel. E) Application of the vascularized tumor chip for evaluating the promoted transport of anticancer drugs, immune cells, and anticancer drug‐conjugated liquid metal (embolism materials).

We intended to induce angiogenic sprouting from mature straight blood vessels by controlling the flow conditions and the existence of stromal cells. A straight blood vessel was fabricated using microneedles as the template.^[^ ^45^ ^]^ The microfluidic chip comprised a hydrogel part, where three parallel microchannels were formed within the hydrogel, and the polydimethylsiloxane (PDMS)‐based housing part, in which six reservoirs were located. The hydrogel comprised collagen type I and fibrin. Collagen type I is abundant in the lung tissue and around the tumor^[^ ^46^ ^]^ and provides mechanical support for the formation of collagen microchannels. Fibrin is known to induce vasculogenesis and angiogenesis.^[^ ^47^, ^48^ ^]^ The appropriate ratio between collagen type I and fibrin was determined considering the angiogenic capabilities. The diameter of the vascular channel and the hollow channel was 235 µm, and the human lung fibroblast (hLF)‐tumor spheroid channel had a diameter of 550 µm, considering the size of the tumor spheroid (**Figure** **1**A). Human umbilical vein endothelial cells (HUVECs) were used as model cells for endothelial cells because of their high angiogenic potential. The late incorporation of lung cancer spheroids is desirable as the early interaction of cancer cells and endothelial cells, before the maturation of microvasculature, can lead to a disrupted blood vessel.^[^ ^49^, ^50^ ^]^ The angiogenesis of HUVECs was compared depending on i) the flow direction, ii) existence of hLFs, and iii) location of tumor spheroids, focusing on the sprouting direction and speed.^[^ ^51^, ^52^ ^]^

**Figure 2:**
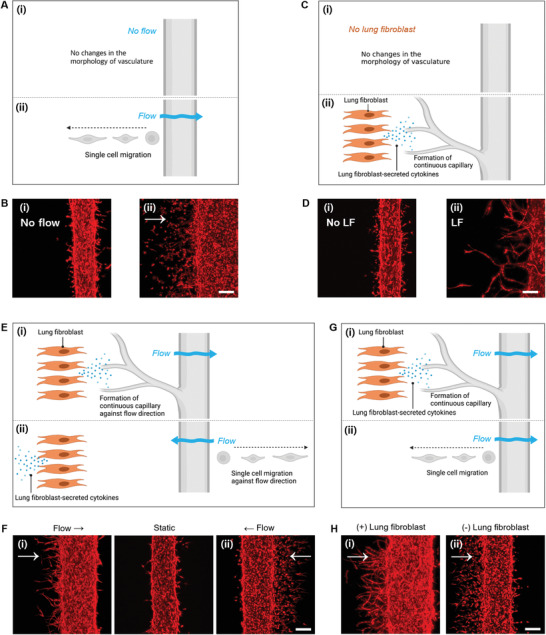
Control of angiogenesis depending on flow direction and existence of human lung fibroblasts. A,B) Effect of flow in the migration of endothelial cells. Under this condition (flow‐only condition), flow was applied from left to right in the absence of human lung fibroblasts, and endothelial cells migrated as single cells against the flow (day 5). C,D) Effect of human lung fibroblasts in the growth pattern of the capillary. Under this condition (lung fibroblast‐only condition), no flow was induced and lung fibroblasts were present in the left‐side channel (day 5). E,F) Synergistic effect of flow direction and lung fibroblast existence in angiogenesis. E) Schematic of the behaviors of endothelial cells in the presence of fluid flow and human lung fibroblasts. F) Growth patterns of RFP‐expressing HUVECs on day 4 depending on the flow directions. G,H) Effect of fibroblast presence on continuous capillary formation. G) Schematic of the behaviors of endothelial cells in the presence of human lung fibroblasts. H) Continuity and lumen formation of capillaries depending on the presence and location of human lung fibroblasts on day 5. Scale bar: 200 µm.

First, the direction of interstitial flow on capillary sprouting was evaluated. The direction of the interstitial flow within the composite hydrogel was controlled by setting the volume differences between the channel reservoirs. For example, by increasing the volume in the reservoirs of the hLF channel or decreasing that in the reservoirs of the hollow channel, an hLF channel‐to‐hollow channel interstitial flow was generated. We found that the direction of angiogenic sprouting was opposite to the flow direction. When there was no interstitial flow, the HUVECs did not show any preferred sprouting or migration to either side (**Figure** **2**A,Bi). Such flow‐direction‐dependent angiogenesis has been confirmed in previous studies.^[^ ^29^ ^]^ This evidently shows that the direction of interstitial flow controls the sprouting direction of the capillaries (Figure 2A,Bii).

Second, hLF regulates lumen formation in sprouted capillaries. This difference is observed in the presence of interstitial flow. When hLFs existed in the hLF channel, the sprouted capillary from the mature main microvasculature showed continuous forms (Figure 2C,Dii), which was different from the single‐cell migration behaviors in the absence of hLFs (Figure 2C,Di). The position of hLFs is also important for the continuity of angiogenesis. When the hLFs were located in the left channel, the flow from left to right induced continuous capillary formation with a lumen structure (Figure 2E,Fii); however, the flow from right to left caused single‐cell migration (Figure 2E,Fi). These results clearly show that the hLF‐secreted factors are delivered along with the flow and ultimately regulate the continuity and lumen formation of newly formed capillaries (Figure 2G). Thus, by decoupling the flow direction and the existence of hLFs, we confirmed their individual roles in angiogenesis.

### 2.2 Growth Pattern of Microvessels Depending on the Presence of Each Factor

**Figure 3:**
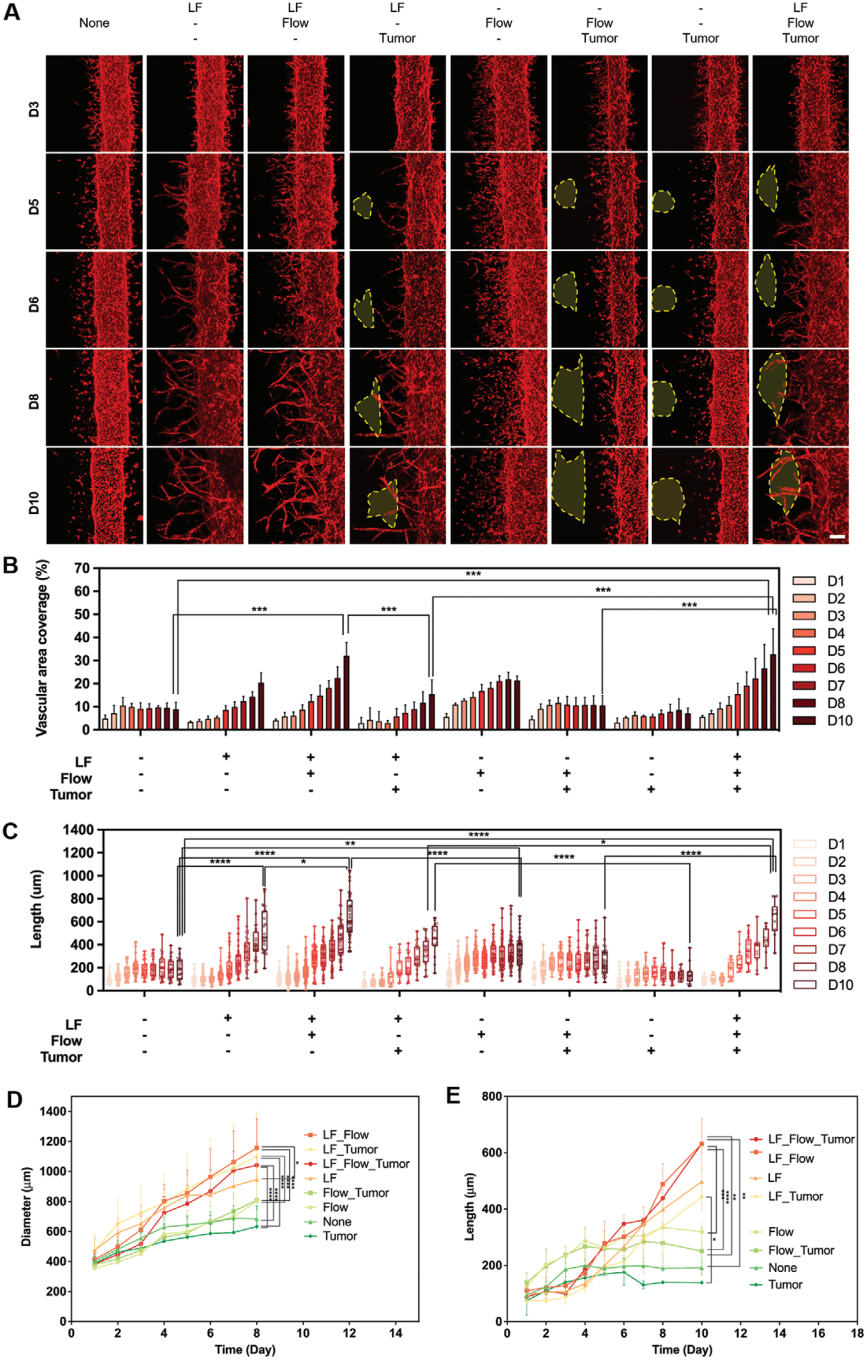
Screening efficient angiogenesis conditions depending on the combination of three experimental factors: flow, hLFs, and tumor. A) Growth of blood vessels depending on the presence or absence of fibroblasts, flow, and tumor spheroids. Scale bar: 200 µm. Yellow dotted line denotes tumor spheroids. B) Quantification of vascular area coverage depending on the combination of the three experimental factors (***p < 0.001). C,D) Growth rate of microvascular length depending on the combination of the three experimental factors (*p < 0.05, **p < 0.01, ***p < 0.001, and ****p < 0.0001). E) Changes in the diameter depending on the combination of the three experimental factors (*p < 0.05, **p < 0.01, ***p < 0.001, and ****p < 0.0001).

We utilized red fluorescent protein (RFP)‐expressing HUVECs to monitor the time‐dependent growth pattern of capillaries from mature microvasculature. We tracked the individual chips for 10 days in a live state using a confocal microscope, and the growth pattern and speed of the capillary were investigated depending on the combination of three factors: existence of hLFs, interstitial flow, and presence of tumor spheroids (**Figure** **3**A).

The vascular coverage area was highly promoted when both hLFs and flow were present (Figure 3A and B). Notably, when comparing the hLF‐only and flow‐only conditions, the former induced a monotonic increase in coverage area, whereas the latter exhibited an increase in the coverage area at the later stage of sprouting, showing an increase in the slope gradient (Figure 3B). The presence of hLFs induced angiogenesis toward them. As shown in Figure 3A, the existence of hLFs on the left side of the main blood vessel induced continuous capillary growth toward the left side in the absence of flow. This hLF‐induced capillary growth is mediated by the diffusion of hLF‐secreted factors.^[^ ^51^ ^]^ The existence of tumor spheroids showed no notable difference in vascular area coverage.

The sprouting time length showed a similar pattern, and was the longest when hLFs and flow coexisted (Figure 3C). Considering the individual contribution of hLFs and flow, the hLF‐only condition showed a longer sprouting time than the flow‐only condition. The existence of tumor spheroids induced no difference in capillary sprouting time length; however, the growth direction was affected by the tumor spheroids in the early stages (days 4–5) (Figure 3A).

In addition, by comparing the diameter of the main microvasculature under each condition, the diameter was more than doubled when hLFs were present (Figure 3D). The effect of flow was smaller than that of hLFs (Figure 3E); however, the diameter increased with time (Figure 3D).

In the flow + tumor condition, the diameter of the main blood vessel was smaller than that in the other conditions. Tumor spheroids induce the growth of blood vessels by secreting proangiogenic factors, but A549 cells are known to have limited tumor angiogenesis capability on their own.^[^ ^35^ ^]^ Furthermore, some studies have shown that the existence of tumor spheroids near blood vessels causes vascular inflammation and leaky blood vessels.^[^ ^53^ ^]^ Furthermore, tumor cells are known to retard or degrade vascularization in a tumor‐vasculature coculture model.^[^ ^54^ ^]^ Although various factors may contribute to angiogenesis, we presume that the tumor‐secreted factors delivered by flow can suppress the growth and maturation of the main blood vessel. In the tumor‐only condition, the main blood vessel grew, suggesting the importance of flow‐mediated delivery of soluble factors in blood vessel growth and maturation.

### 2.3 Morphological Analysis of the Vascularized Lung Cancer Model

**Figure 4:**
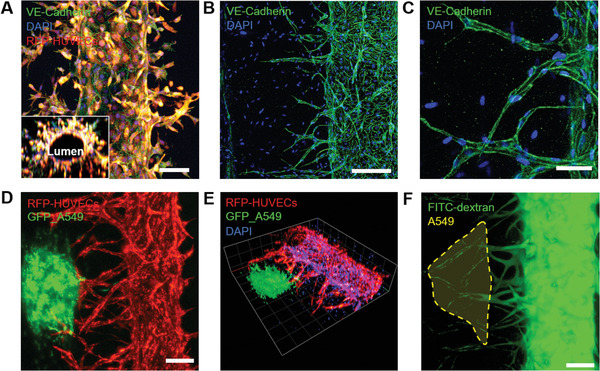
Immunofluorescence images of the engineered 3D microvasculature. A) Fluorescence images of the engineered blood vessel (red: RFP‐expressing HUVECs; green: VE‐cadherin; blue: DAPI). i) RFP‐expressing HUVECs formed adherens junctions of VE‐cadherin. A 3D reconstructed image of the cross‐section shows the hollow lumen structure. Scale bar: 100 µm. ii) Confocal microscopic image of angiogenic sprouts grown from the main HUVEC microvasculature. Scale bar: 200 µm. iii) Enlarged view of the sprouted capillaries showing VE‐cadherin expression. Scale bar: 50 µm. B) Fluorescence images of the vascularized lung cancer model. i) Z‐projection of the 3D vascularized tumor model (red: RFP‐HUVEC and green: GFP‐A549). Scale bar: 200 µm. ii) A 3D reconstructed view of the vascularized lung cancer model (red: RFP‐HUVEC, green: GFP‐A549, and blue: DAPI). iii) Vascularized tumor system perfused with 40 kDa FITC‐labeled dextran. Scale bar: 200 µm.

To confirm the 3D structural shape of the blood vessels cultured in the chip and visualize the characteristic marker expression of the blood vessels, z‐stack images were obtained. Cross‐sectional images of blood vessels showed that HUVECs formed a lumen and maintained their tubular shape in the composite hydrogel (**Figure** **4**A). Both RFP‐expressing HUVECs and normal HUVECs expressed vascular endothelial (VE)‐cadherin at the cellular junctions (Figure 4Ai,ii). Specifically, we confirmed the expression of VE‐cadherin in capillaries that grew out of the main blood vessels (Figure 4Aiii). When a tri‐culture of tumor spheroids, HUVECs, and hLFs was studied under the previously selected conditions, a vascularized tumor model was observed on the chip on day 8 (Figure 4Bi,ii). The perfusion and formation of lumen structures were visualized by introducing a 40 kDa fluorescein isothiocyanate (FITC)‐dextran solution through the main blood vessel (Figure 4Biii). FITC‐dextran was observed throughout the capillary structure, confirming the formation of the perfusable capillary structure by the sophisticated engineering of the microenvironment around the blood vessel and tumor spheroids. This model can be used to evaluate drug delivery and efficacy.

### 2.4 Evaluation of Drug Delivery Using the Vascularized Lung Cancer Model

**Figure 5:**
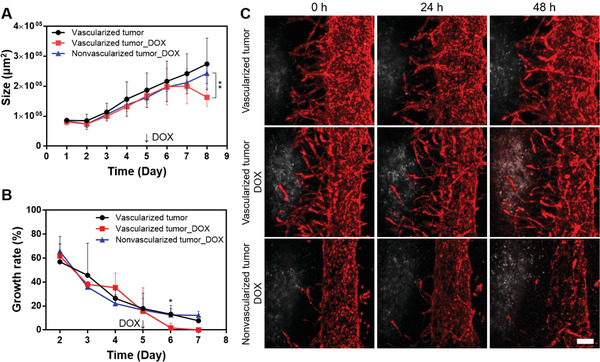
Effect of DOX treatment in the arrest of tumor growth, and drug delivery of DOX through the sprouted capillaries. A) Effect of vascularization on tumor size upon DOX treatment (**p < 0.01). B) Time‐dependent growth rate of vascularized and nonvascularized tumors after DOX treatment (*p < 0.05: Vascularized tumor versus Vascularized tumor_DOX). C) Confocal microscopy images of DOX‐treated tumors and vasculature depending on the exposure time (red: RFP‐HUVEC, white: A549). Scale bar: 200 µm.

We performed a drug delivery experiment using an engineered vascularized lung cancer model. The anticancer drug doxorubicin‐HCl (DOX) was used to treat vascularized and nonvascularized tumor spheroids, and the growth rate of the tumor spheroids was analyzed. The interstitial flow was stopped during exposure to DOX, immune cells, and DOX‐conjugated liquid metal particles (embolism materials). DOX, immune cells, and embolism materials were introduced through the main blood vessels by mixing with the cell culture medium. In both vascularized and nonvascularized cases, DOX was delivered through the main blood vessels. The nonvascularized model represents the coculture model of tumor spheroids, hLFs, and HUVECs without the induction of angiogenic sprouting, and thus, the main microvasculature had a straight morphology without branches. In the DOX‐treated vascularized tumor model, the difference was not statistically significant before DOX treatment on days 5 and 6. However, after 72 h of drug treatment, there was a significant difference in size between vascularized and nonvascularized tumors, with only the vascularized tumor showing a reduction in tumor size (**Figure** **5**A). The growth of the DOX‐treated vascularized tumor stopped on day 7, suggesting arrested growth due to DOX (Figure 5B). In Figure 5C, RFP‐HUVECs (red) and A549 tumor spheroids (white) are displayed. When DOX was perfused through the main blood vessel, nonvascularized tumors (bottom row) showed limited DOX signals (red) overlapped with tumor spheroids (white), while vascularized tumors (middle row) showed a DOX signal overlapped with A549 tumor spheroids (white). The overlapped signal indicates that DOX was delivered to the tumor spheroids. This is consistent with the reduced tumor size shown in Figure 5A,B. Furthermore, in the vascularized tumor model, the amount of DOX uptake in tumor spheroids increased, suggesting a more efficient delivery of anticancer drugs in the proximity of tumor spheroids through the sprouted capillaries (Figure 5C).

### 2.5 Transport Assay of Immune Cells Through the Sprouted Capillaries

**Figure 6:**
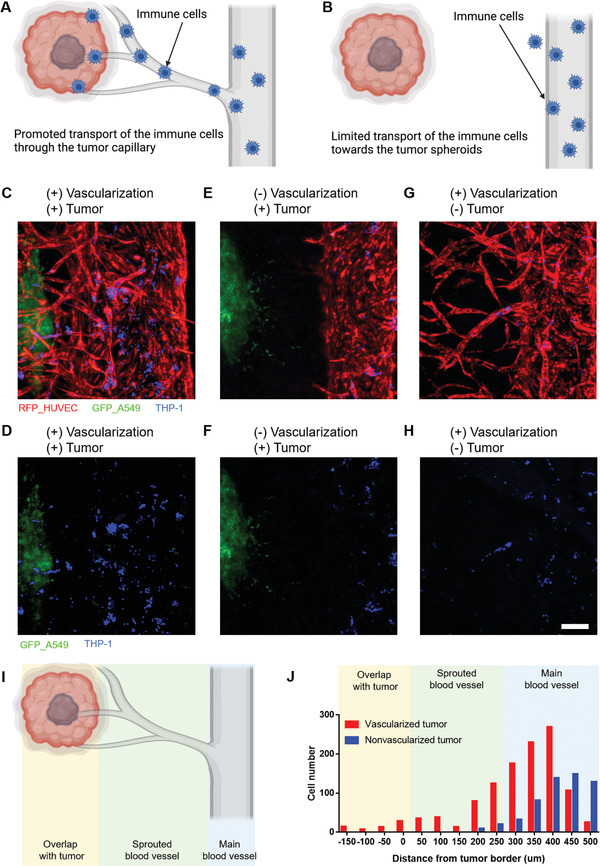
Effects of vascularization in the delivery of immune cells (THP‐1) to the tumor spheroids. A) Schematic of a vascularized tumor case in which the transport of immune cells is promoted through the capillary. B) Schematic of a nonvascularized tumor case in which the transport of immune cells is limited. C,D) When the THP1 cells were delivered to the tumor spheroid through the microvessels on the vascularized tumor chip, many of the THP1 cells were observed near the tumor spheroid (red: RFP‐HUVEC, green: GFP‐A549, and blue: THP1). E,F) When the THP1 cells were introduced through the main blood vessel, cells were barely observed near the tumor spheroids due to the limited cell transport. G,H) When the THP1 cells were treated on the blood vessel‐only chip, few cells were observed in the sprouted capillaries. Scale bar: 200 µm. I) Schematic of regions analyzed for immune cell transport. J) Cell number in each region in the cases of vascularized and nonvascularized tumors.

Because this platform has perfusable capillary structures, it can be used to monitor the transport of immune cells through tumor‐associated blood vessels. Ultimately, it can be applied to evaluate the efficacy of cancer immunotherapy. THP1, a progenitor macrophage cell line, was used as a model immune cell line. THP1 cells were labeled with Cell Tracker Blue and introduced into the main blood vessel.^[^ ^55^ ^]^ We performed experiments on vascularized tumor spheroid chips, vascular‐only chips, and nonvascularized tumor spheroid chips. The THP1 cells (labeled in blue) migrated through the capillaries in the vascularized tumor and vasculature‐only cases (**Figure** **6**C,D) and (Figure 6G,H). Specifically, in the vascularized tumor model, THP1 cells arrived at the proximity of the tumor spheroid along the sprouted capillaries (Figure 6C,D). In the nonvascularized tumor microfluidic chip, THP1 cells were present only in the main vascular channel (Figure 6E,F), showing limited access of immune cells to the tumor spheroid. The quantified cell numbers in each region, such as the overlap with the tumor, sprouted blood vessel, and main blood vessel regions, indicated that the immune cells could be efficiently transported toward the tumor spheroids (Figure 6I,J). The cell number under “overlap with the tumor region” considered the total yellow region. During the analysis, we found that the number of immune cells in vascularized tumors was consistently larger than that in nonvascularized tumors. We speculated that other factors may contribute to the large number of remaining immune cells in the vascularized tumors. As shown in Figure 6J, in the main blood vessel zone (blue), immune cells are prone to be localized on the left side of the blood vessel that is close to the vascularized region. Such an asymmetric distribution is predominantly regulated by the flow toward the tumor through the vascularized capillary. These results indicate that the vascularized tissue model can be used for studies on cancer immunotherapy using cytotoxic immune cells such as T cells or natural killer (NK) cells.

### 2.6 Embolism of Tumor Capillaries with DOX‐Conjugated Liquid Metal Particles

**Figure 7:**
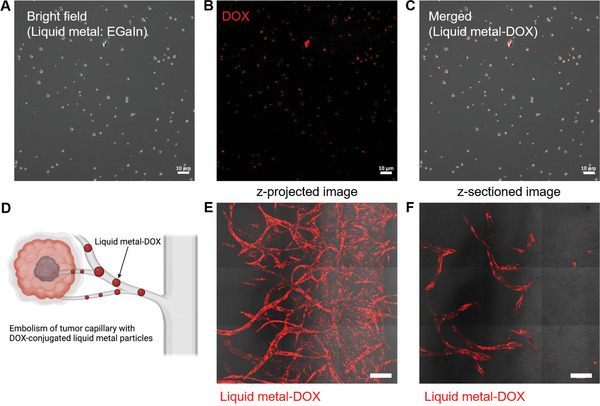
Embolism of tumor capillaries with DOX‐conjugated liquid metal particles. A) Bright‐field microscopic image of DOX‐conjugated liquid metal (EGaIn) particles. B) Fluorescence image of DOX conjugated with liquid metal particles. C) Merged images of bright‐field and fluorescence images of DOX‐conjugated liquid metal particles. Scale bar: 10 µm. D) Schematic of the embolism strategy for tumor capillaries with DOX‐conjugated liquid metal particles. E) Merged image of bright‐field and fluorescence signals after the introduction of liquid metal‐DOX through the main blood vessel (z‐projected image). F) Images of liquid metal‐DOX under the flow condition along the main blood vessel (z‐sectioned image). Scale bar: 200 µm.

The vascularized tumor platform can be used to test whether the embolism strategy can be used to target tumor capillaries. Thus, we utilized DOX‐conjugated liquid metal (eutectic gallium‐indium (EGaIn)) particles. Bright‐field and fluorescence images showed the colocalization of the liquid metal nanoparticles and DOX (**Figure** **7**A–C). Owing to their small size, liquid metal particles can be transported through the capillary via simple loading at the reservoirs. Furthermore, they can not only aggregate and transform their structure via external stimuli, such as heat, but also act as carriers for the transportation of anticancer drugs (Figure 7D).^[^ ^56^ ^]^ These DOX‐conjugated liquid metal particles were introduced through the main blood vessel via gravity‐driven flow (Figure 7E). Liquid metal‐DOX was successfully delivered to the capillary (Figure 7F). The z‐sectioned image showed that the liquid metal‐DOX remained in the capillary even under flow conditions, suggesting a successful embolism of the tumor capillaries. However, the decrease in tumor spheroids could not be tested because the culture media surrounding the tumor spheroid readily possessed sufficient levels of metabolic substances even after the embolism.

## 3 Discussion

We presented a 3D vascularized tumor model that spontaneously sprouted capillaries toward the tumor spheroid with the help of controlled interstitial flow and hLF‐secreted factors. The main microvasculature was attached to HUVECs on the luminal surface of the pre‐formed microchannels.^[^ ^57^ ^]^ In this platform, the lung cancer spheroid was introduced on day 4; thus, it did not affect the vascular maturation stage. The hLFs and tumor spheroids were located at a defined distance from the microvasculature. This allows for the distant sprouting of capillaries through the cell‐free hydrogel matrix and long‐range interaction between the blood vessel and hLFs, other blood vessels, and tumor spheroids.

Microfluidic chips with micropillar arrays are widely used as alternative platforms. In this system, hydrogels were patterned using a high capillary pressure between the micropillars; thus, easy and reproducible production was possible. This system also provided a 3D cell culture environment, and various vascularized tumor models were also presented. However, this system has a heterogeneous interface at the PDMS–glass bonding and a nonreconfigurable vascular structure, owing to the rigid materials around the blood vessel.^[^ ^21^, ^35^, ^44^ ^]^ Attempts have also been made in the past to form vascular networks with various ECM scaffolds using 3D bioprinting.^[^ ^42^, ^58^ ^]^ Based on this, cancer intravasation or angiogenic sprouting models are being studied; however, compared to other platforms, they are relatively expensive and require skills such as uniform cell seeding and temperature‐sensitive matrix control.^[^ ^59^, ^60^, ^61^ ^]^ We adapted fabrication methods using multiple microneedles as templates.^[^ ^41^, ^62^ ^]^ In this platform, the blood vessel was embedded in the bulk hydrogel, and thus, the surrounding 3D environment was homogeneous and could be remodeled by the cells. Furthermore, the direction and speed of the interstitial flow can be controlled by regulating the height difference of the medium in each reservoir.

Various factors are known to induce angiogenesis, including interstitial flow,^[^ ^51^ ^]^ fibroblasts,^[^ ^63^ ^]^ tumor cells,^[^ ^40^ ^]^ and growth factors.^[^ ^37^, ^41^ ^]^ In this study, the effects of flow, fibroblast, and tumor spheroid were examined, and the proangiogenic combination of the conditions was investigated.^[^ ^64^ ^]^ According to the study findings, fibroblast‐induced factors are essential for lumen formation, and the direction of flow determines the direction of the initial sprouting of microvessels. The presence of tumor spheroids resulted in a tendency for blood vessels to sprout toward it; however, such an effect was minor, likely due to the relatively less aggressive phenotype of A549 cells.^[^ ^35^ ^]^ Some studies reported spontaneous angiogenesis only in the presence of tumor cells without the help of interstitial flow. Miller et al. used 10T1/2 cells, a mouse embryo fibroblast cell line derived from sarcoma,^[^ ^65^ ^]^ and Nagaraju et al. utilized MDA‐MB‐231 and MCF‐7 human breast cancer cell lines.^[^ ^66^ ^]^ However, in our study, we used the A549 human lung cancer cell line. Nashimoto et al. showed that A549 cells alone cannot induce angiogenesis.^[^ ^35^ ^]^ Considering these findings, it seems that cancer cell‐induced angiogenesis is dependent on the angiogenic capability of the cancer cells.

The vascularized tumor platform was used to evaluate the delivery and efficacy of anticancer drugs in tumor spheroids. Tumor spheroid growth did not significantly differ in the presence or absence of sprouted capillaries up to 48 h after treatment with 5 µg mL^−1^ DOX. However, after 72 h of exposure to DOX, the tumor size decreased in the vascularized tumor model because of the increased DOX delivery through the sprouted capillaries. Furthermore, increased uptake of DOX was observed in vascularized tumor spheroids. Although the vasculature was damaged by DOX, the vascularized tumors showed higher drug transport to the tumor spheroids because the sprouted capillaries reduced the transport distance. In the results of previous studies, the drug was used at such a low concentration that did not affect blood vessels, and the effect of oxygen and nutrient delivery through blood vessels was more significant than the effect of the drug.^[^ ^35^ ^]^ Therefore, this platform can be considered more suitable for the screening of anticancer drugs that do not disrupt blood vessels and drugs that cotarget both tumor and blood vessels.

Preliminary experiments were conducted to evaluate the transport of THP‐1 cells to the tumor spheroid through the sprouted capillaries. This approach can potentially be applied to cancer immunotherapy. The results showed that THP‐1 cells were more profoundly observed near the tumor spheroids, suggesting the contribution of vascular structure in the delivery of immune cells.

The blocking or ablation of tumor capillaries is a promising strategy to arrest the growth of solid tumors. Our vascularized platform can evaluate whether a material can block the tumor capillaries. The liquid metal‐DOX could be delivered through the capillary via simple loading and remained even under perfusion conditions. However, the death of tumor cells or size reduction of tumor spheroids could not be demonstrated because growth factor‐ and fetal bovine serum‐depleted media could not be selectively delivered around the tumor spheroids. If a region‐specific starvation condition can be established, embolism of the tumor capillaries is expected to reduce the tumor size.

## 4 Conclusions

In this study, we demonstrated the development of vascularized lung cancer models through the sophisticated control of the microenvironment. The interstitial flow direction and the existence of hLFs contributed individually to capillary sprouting and lumen formation, respectively. Although the effect of cancer cells was limited in our model cell, A549, such an effect may differ depending on the tumor cell type. Using the vascularized lung cancer platform, we demonstrated that the drug delivery efficacy and regression of tumor spheroids were increased in the presence of sprouted capillaries. Furthermore, we also show that neo‐vessels can act as a transport route for immune cells and that they can be blocked by liquid metal nanoparticles. This vascularized tumor platform could potentially be used in the screening of anticancer drugs cotargeting tumors and blood vessels, or in the evaluation of the efficacy of anticancer therapeutics.

## 5 Experimental Section

### Device Fabrication

The microfluidic device was fabricated using a metal frame and microneedles as templates. A 10:1 (w/w) mixture of PDMS (Sylgard 184, Dow Corning, MI, USA) was poured onto the metal frame with the needle inserted and degassed under vacuum. The assembly of the PDMS‐filled metal frame and glass was cured for 1 h in an oven at 80 °C. After the separation of the frame and glass substrate, the microneedles were removed from the cured PDMS. The part of the hydrogel for injection was cut out with a 4 × 5 mm square punch, and subsequently, a flat PDMS sheet was bonded with the punched PDMS after oxygen plasma treatment. The reservoirs for the cell culture medium and the holes for the hydrogel injection were punched out using 8 and 1 mm biopsy punches (Kai Medical, Tokyo, Japan), respectively. After inserting the needle into the PDMS chip, dust was removed using a residue‐free tape, and the PDMS chip was assembled with a cover glass (50 × 70 mm, Matsunami Glass Inc., Osaka, Japan) after treatment with oxygen plasma (FEMTO Science, Hwaseong, South Korea) (Figure 1A). UV irradiation was performed before the experiment to ensure sterilization.

### Cell Culture

HUVECs (Lonza, Basel, Switzerland) and RFP‐expressing HUVECs (Angio‐Proteomie, Boston, MA, USA) were cultured in endothelial cell medium (ScienCell, Carlsbad, CA, USA), and cells from passages 2–4 were used for the experiments. Normal hLFs (Lonza, Basel, Switzerland) were cultured in fibroblast growth medium (FGM‐2, Lonza, Basel, Switzerland), and cells from passages 3–5 were used for the experiments. THP‐1 (KCLB, Seoul, South Korea) and A549 (KCLB, Seoul, South Korea) cells were cultured in Roswell Park Memorial Institute (RPMI)‐1640 medium (Welgene, Gyeongsan, Korea) supplemented with 10% fetal bovine serum and 1% (v/v) penicillin–streptomycin. Green fluorescent protein (GFP)‐expressing A549 cells (MyBioSource, San Diego, CA, USA) were cultured in Dulbecco's modified Eagle's medium (DMEM, Welgene, Gyeongsan, South Korea) supplemented with 10% fetal bovine serum and 1% (v/v) penicillin–streptomycin. All cells were maintained in a humidified incubator at 37 °C with 5% CO_2_.

### Cell Seeding in the Devices

To ensure adhesion between the composite hydrogel and the PDMS device, a 2 mg mL^−1^ dopamine chloride (Sigma‐Aldrich, St. Louis, MO, USA) solution was added in the dark for 2 h. The composite hydrogel was prepared by dissolving the following substances in DMEM: 2.8 mg mL^−1^ fibrinogen (Sigma‐Aldrich, St. Louis, MO, USA), 0.3 µg mL^−1^ aprotinin (Sigma‐Aldrich, St. Louis, MO, USA), 0.3 mg mL^−1^ collagen type 1 (Corning, NY, USA), and 0.5 U mL^−1^ thrombin (Sigma‐Aldrich, St. Louis, MO, USA). The mixture was immediately injected into a square‐shaped hydrogel chamber through the injection hole. After gelation, the needles were removed, resulting in hollow channels inside the hydrogel (Figure 1B). Afterward, 1.5 × 10^4^ HUVECs were seeded in the middle channel and flipped after 5 min for the homogeneous attachment of seeded cells. Subsequently, 6.0 × 10^3^ hLFs were seeded in a 550 µm diameter channel, tilted at 90°, and incubated for 10 min for the preferred attachment of hLFs on the sidewall (Figure 1C). The interstitial flow direction was adjusted using the gradient of the medium, and the static state was maintained. For the induction of interstitial flow, more medium was applied in the hLF‐seeded channel reservoirs (≈500 µL) than in the other reservoirs (≈100 µL). The maintenance time of the flow condition increased as the culture time increased. In the early stage of culture (DIV 1–5), the flow was maintained for approximately 2–8 h, while in the later stage with tumor spheroids (DIV 6–9), the flow was maintained for more than 24 h due to the growth of cells.

### Preparation of Lung Cancer Spheroids

A549 (lung cancer cell line) and GFP‐expressing A549 cells were used to prepare the tumor spheroids. Each cell was suspended in 7 mL of medium and placed in a SpheroFilm (INCYTO, Cheonan, Korea). After 10 min, the supernatant was removed according to the manufacturer's instructions. RPMI‐1650 and DMEM were used to maintain the tumor spheroids for 4–5 days. The tumor spheroids embedded in the sol‐state composite hydrogel were introduced through the 550 µm diameter channel of the microfluidic chip after the maturation of the blood vessel (4 days after seeding).

### Immunofluorescence Imaging

Cells cultured in the microfluidic chip were fixed with 4% paraformaldehyde (Biosesang, Seongnam, South Korea) for 30 min at 25 °C. Subsequently, the fixed chip was treated with a solution containing 0.1% Triton X‐100 (Sigma‐Aldrich, St. Louis, MO, USA) and 3% bovine serum albumin (Sigma‐Aldrich, St. Louis, MO, USA) dissolved in phosphate‐buffered saline (PBS) at 25 °C for 1 h for permeabilization and blocking. For the staining of adherens junctions, anti‐VE‐cadherin (Santa Cruz Biotechnology, Dallas, TX, USA) was incubated overnight at 4 °C, followed by incubation with Alexa Fluor 488 (Invitrogen, Waltham, MA, USA) at 25 °C for 2 h. DNA was stained with 4′,6‐diamidino‐2‐phenylindole (DAPI, Thermo Fisher Scientific, Waltham, MA, USA) and incubated at 25 °C for 30 min. To obtain 3D sectional images, the stained samples were examined using a confocal laser scanning microscope (LSM 700, Carl Zeiss, Oberkochen, Germany).

### Evaluation of Microvascular Network Perfusion

FITC‐dextran (Sigma‐Aldrich, St. Louis, MO, USA) was used to confirm the perfusability of the microvascular vessels that had grown toward the tumor spheroid. After the induction of controlled angiogenesis, a 10 × 10^−6^ m FITC‐dextran solution was introduced into the microvasculature channel. Fluorescence images were obtained at 1 min intervals for 5 min.

### Efficacy Testing of Anticancer Drugs

Vascularized and nonvascularized lung cancer microfluidic chips were prepared to investigate drug delivery and the effect of anticancer drugs on cancer regression. On the eighth day (when the tumor spheroids were transferred and cultured for four days in the blood vessel chip), DOX (Sigma‐Aldrich, MO, USA) was introduced into the microvasculature channel at a concentration of 5 µg mL^−1^. Fluorescence images were obtained 24 and 48 h after DOX treatment using an inverted microscope (Olympus, Tokyo, Japan). The size of the tumor spheroids was measured using ImageJ software (National Institutes of Health, Bethesda, MD, USA). For visualization of vascular disruption, samples were treated with DOX for 24 h, and after vascular recovery, they were fixed and immunostained with VE‐cadherin. Immunostained samples were examined using a confocal laser scanning microscope (LSM 700).

### Transport Assay of Immune Cells

After harvesting THP‐1 cells from the cell culture flask, the cell pellet was resuspended in RPMI‐1640 medium. The cell tracker (Invitrogen, Waltham, MA, USA) was added to the cell suspension and incubated for 30 min under growth conditions. After centrifugation and removal of the supernatant solution, fresh medium was added. Afterward, 4.0 × 10^4^ cell tracker‐labeled THP‐1 cells were introduced into each chip: microfluidic chips with a vascularized tumor spheroid, microfluidic chips with only microvascular vessels, and microfluidic chips with a nonvascularized tumor spheroid. After 24 h, the locations of THP‐1 cells were examined using a confocal microscope (LSM 700). For the immune cell transport assay, immune cells were first introduced into the main blood vessel and the same flow as that for DOX treatment was applied. The flow was maintained for 24 h, and the number of remaining immune cells was counted.

### Quantification of Blood Vessel Area and Length and Diameter of Sprouts

To quantify the vascular area and length of sprouts depending on the experimental conditions, z‐stack images were obtained daily with a confocal microscope (LSM 700). To analyze the vascular area, the fluorescence signals in the region of interest were measured from the edge of the main vascular channel using ImageJ software. The length of the capillary sprouted from the main vascular wall was quantified using ImageJ software. Bright‐field images were captured using an inverted microscope (Olympus, Tokyo, Japan) to quantify the diameter of the main vessel for each condition, and the diameters were measured using ImageJ software.

### Embolism of Tumor Capillaries with DOX‐Conjugated Liquid Metal Nanoparticles

DOX‐conjugated liquid metal nanoparticles were prepared according to a previously described protocol.^[^ ^56^ ^]^ Briefly, 10 mg of EGaIn, 80 µL of distearoylphosphatidylcholine (25 mg mL^−1^ in chloroform), and 400 µL of 1,2‐distearoyl‐*sn*‐glycero‐3‐phosphoethanolamine‐*N*‐[amino(polyethylene glycol)‐2000] (25 mg mL^−1^ in chloroform) were added to a 50 mL disposable vial. Sonication (2501E‐DTH, Branson Ultrasonics, Brookfield, CT, USA) was applied to the vial for 10 min at 50 °C. A powder was obtained after removing the chloroform from the vial by placing it in a dry oven. Deionized (DI) water (10 mL) was added to the powder and mixed. The mixture was dispersed for 1 h using probe sonication (VCX 750, Sonics & Materials, Newtown, CT, USA) with an amplitude of 26% and a pulse of 5 s on/5 s pause. The solution was centrifuged at 15 000 × *g* for 10 min, washed twice with 10 mL of DI water, and left for 1 h to remove large particles via sedimentation. For DOX conjugation in liquid metal, 4 mg of DOX was dissolved in 4 mL of dimethyl sulfoxide, 2 µL of triethanolamine was added, and the mixture was incubated at 25 °C for 12 h. After the reaction, DOX (1 mg mL^−1^) was added to the sonicated sample particles so that the ratio of EGaIn to DOX was 10:1, and reacted at 4 °C for 12 h on the rocker. After the reaction, the samples were centrifuged at 15 000 × *g* for 10 min to remove free DOX and then rehydrated in 10 mL of DI water or 1× Dulbecco's phosphate‐buffered saline (DPBS, pH 7.5). For embolism of the tumor capillary, 50 µL of the prepared solution was introduced into the main blood vessel and allowed to stand for 1 h. Gravity‐driven flow was induced by balancing the volume of the cell culture medium without the liquid metal in each reservoir, and the flow was maintained for 24 h. After 24 h of flow exposure, fluorescent and bright‐field images were captured using a confocal microscope (LSM 700).

### Statistical Analysis

All experiments were repeated at least three times, and the data were analyzed using Prism software (GraphPad Software, San Diego, CA, USA). Statistical comparisons of analyzed values were obtained from one‐way ANOVA and Tukey's HSD test, with the *p*‐value threshold for statistical significance set at *p < 0.05, **p < 0.01, ***p < 0.001, ****p < 0.0001, and ns (not significant) at *p* > 0.05.

## Conflict of Interest

The authors declare no conflict of interest.

## Data Availability

The data that support the findings of this study are available on request from the corresponding author. The data are not publicly available due to privacy or ethical restrictions.
